# How common and frequent is heterosexual anal intercourse among South Africans? A systematic review and meta-analysis

**DOI:** 10.7448/IAS.20.1.21162

**Published:** 2017-01-11

**Authors:** Branwen N. Owen, Jocelyn Elmes, Romain Silhol, Que Dang, Ian McGowan, Barbara Shacklett, Edith M. Swann, Ariane van der Straten, Rebecca F. Baggaley, Marie-Claude Boily

**Affiliations:** ^a^ Department of Infectious Disease Epidemiology, Imperial College London, London, UK; ^b^ Vaccine Clinical Research Branch, Division of AIDS, National Institutes of Allergy and Infectious Diseases, US National Institutes of Health, Bethesda, MD, USA; ^c^ Department of Medicine, University of Pittsburgh School of Medicine, Pennsylvania, UK; ^d^ Department of Medical Microbiology and Immunology, University of California, Davis, CA, USA; ^e^ Women’s Global Health Imperative Program, RTI International, San Francisco, CA, USA; ^f^ Department of Infectious Disease Epidemiology, London School of Hygiene and Tropical Medicine, London, UK

**Keywords:** Anal intercourse, heterosexual, sexual behaviour, South Africa

## Abstract

**Background**: HIV is transmitted more effectively during anal intercourse (AI) than vaginal intercourse (VI). However, patterns of heterosexual AI practice and its contribution to South Africa’s generalized epidemic remain unclear. We aimed to determine how common and frequent heterosexual AI is in South Africa.

**Methods**: We searched for studies reporting the proportion practising heterosexual AI (prevalence) and/or the number of AI and unprotected AI (UAI) acts (frequency) in South Africa from 1990 to 2015. Stratified random-effects meta-analysis by sub-groups was used to produce pooled estimates and assess the influence of participant and study characteristics on AI prevalence. We also estimated the fraction of all sex acts which were AI or UAI and compared condom use during VI and AI.

**Results**: Of 41 included studies, 31 reported on AI prevalence and 14 on frequency, over various recall periods. AI prevalence was high across different recall periods for sexually active general-risk populations (e.g. lifetime = 18.4% [95%CI:9.4–27.5%], three-month = 20.3% [6.1–34.7%]), but tended to be even higher in higher-risk populations such as STI patients and female sex workers (e.g. lifetime = 23.2% [0.0–47.4%], recall period not stated = 40.1% [36.2–44.0%]). Prevalence was higher in studies using more confidential interview methods. Among general and higher-risk populations, 1.2–40.0% and 0.7–21.0% of all unprotected sex acts were UAI, respectively. AI acts were as likely to be condom protected as vaginal acts.

**Discussion**: Reported heterosexual AI is common but variable among South Africans. Nationally and regionally representative sexual behaviour studies that use standardized recall periods and confidential interview methods, to aid comparison across studies and minimize reporting bias, are needed. Such data could be used to estimate the extent to which AI contributes to South Africa’s HIV epidemic.

## Introduction

The increased risk of HIV transmission during receptive anal intercourse (AI) compared to receptive vaginal intercourse (VI) is long established, yet its role as a determinant of epidemics driven by sex between men and women (heterosexual sex) in different settings remains uncertain [1–6]. HIV transmission risk during receptive anal intercourse unprotected by condoms (UAI) may be up to 18-fold higher than during receptive VI unprotected by condoms (UVI) [3,7]. Thus, even low frequency of UAI could contribute significantly to HIV transmission among those practising heterosexual sex [8]. The risks of AI have often been omitted from sexual health messaging targeted at people who have heterosexual sex. This has potentially led to the misconception that UAI is safe and may have driven lower condom use during AI among heterosexuals [9–11]. Previous modelling studies also suggest that even a small fraction of UAI could not only influence HIV spread but also reduce the potential impact of specific interventions such as topical vaginal microbicide (VMB) [2,4,8,12]. In contrast, tenofovir, the active pharmaceutical ingredient in oral pre-exposure prophylaxis (PrEP) has been found at higher concentration in rectal than vaginal tissue, suggesting that PrEP may be more protective during receptive AI than VI [13–17].

South Africa is an important setting to examine patterns of heterosexual AI, as it has the largest HIV epidemic driven by heterosexual sex in the world [18]. Its epidemic is among the most researched in sub-Saharan Africa. However, the high prevalence of HIV infection, particularly among young women, and the extent to which AI plays a role, are not well understood [19,20]. Reporting accuracy is also a particular concern for AI compared to VI throughout sub-Saharan Africa not only because, as in many countries worldwide, it is perceived as less socially acceptable and thus liable to underreporting [21–23], but also because local languages refer to AI only in euphemistic terms which are subject to misinterpretation [10,24–26]. Understanding the impact of study characteristics on estimates of AI is critical to inform future study designs to more accurately capture sensitive data. In order to better understand the contribution of AI to South Africa’s HIV epidemic, detailed quantitative information is needed on who practises UAI and how frequently [27].

This paper presents our systematic review of evidence from published literature on self-reported sexual behaviour to determine how common (i.e. how many people) and how frequent (i.e. how often) heterosexual AI and UAI is practised in South Africa. We also describe how AI practices vary by risk group, age, types of partners, setting and over time. This review will be useful to improve our understanding of AI practices, inform prevention messages, and highlight knowledge gaps. Key parameter estimates derived from this review can be used in mathematical models to explore the contribution of AI to the HIV epidemic and assess the influence of AI on the predicted effectiveness of prevention interventions.

## Methods

We conducted this systematic review following MOOSE and PRISMA guidelines [28,29].

### Search strategy

PubMed was searched for English-language articles published January 1990–December 2015 using the search terms ((South Africa OR South African) AND (sexual OR sex) AND (behaviour OR risk) AND (women OR female OR heterosexual)) as MeSH terms (see Section C of the supplementary material for full search terms). The term “anal”, was not included to avoid rejecting studies that, while containing AI data, did not refer to AI in the title or abstract. We first screened titles, discarding those that were obviously irrelevant, then screened abstracts and retrieved full-text articles if any heterosexual sexual behaviour was reported. Full-text articles were screened for quantitative data on AI practices as described below. We scanned the bibliographies of all included articles for further relevant citations. Additionally, we searched for relevant data from national surveys not reported in peer-reviewed journals, which we identified through a Google internet search. We included reports from cross-sectional, cohort and randomized controlled trials (RCTs). Articles which explicitly reported including men who have sex with men (MSM) for which data on heterosexual AI was indistinguishable from homosexual AI, which were conducted wholly or partly outside of South Africa and which did not contain AI data were excluded. We use the term “heterosexual AI” to refer to penile-anal penetrative intercourse with men as the insertive partner and women as the receptive. We refer to participants as being “heterosexual” if they report practising VI with the opposite sex and report no sexual activity with the same sex.

### Data extraction

Our four main outcomes of interest were (i) AI prevalence (the proportion of participants practising AI among sexually active respondents), (ii) monthly frequency of sex acts by type, (iii) fraction of all sex acts and all unprotected sex acts which are AI and UAI, and (iv) fraction of AI and VI acts that are unprotected by condoms. Outcomes were stratified by gender (men, women) where possible (no eligible studies reported any outcome for transgender respondents). When directly reported, we extracted these estimates, otherwise we extracted the relevant information to derive them when available. Thus, we extracted information on the fraction and number of respondents reporting AI and VI intercourse over the various reported recall periods, the mean number of AI, UAI, VI, and UVI acts among those reporting AI and/or the whole sample (i.e. including those who report no AI), the fraction of all sex acts which are AI and UAI, and the fraction of AI and VI sex acts unprotected over each recall period, as well as the 95% confidence intervals (CI) or standard deviation (SD) of each of these, where available. We also extracted information on key participant and study characteristics (gender, survey year, population, mean age, urban or rural and province), including factors reflecting study quality (interview method, study design, sampling method, response rate, survey language and whether heterosexuals only were included). Additionally, we identified the location in the article where AI was first mentioned (title, abstract or main text) and used this to explore publication bias, as papers may report AI behaviour more prominently within the article if the practice is common.

We extracted only baseline data from cohort and RCT studies as we were interested in AI practice in the absence of possible intervention and to minimize potential Hawthorne effect. Where data from the same or overlapping study populations were reported in more than one article, the publication with the largest sample size or with the most information on AI (if the sample size was the same) was included. We contacted authors of included studies when key variables of interest (interview method, recall period for either AI prevalence or frequency or the CI or SD of number of sex acts) were not reported. Samples recruited from communities, schools, health clinics, shebeens (informal drinking establishments) and similar were classified as general-risk populations, while sexually transmitted infection (STI) clinic patients, female sex workers (FSW), their clients, and HIV-infected individuals were classified as higher-risk populations. Relevant information was initially extracted (or derived) into a standard datasheet by BO and double checked by JE. Additional details on methods are provided in supplementary material.

### Data synthesis and statistical methods

#### AI prevalence

Extracted data were used to derive AI prevalence estimates and CIs amongst sexually active participants (defined as those reporting practising VI i.e. the denominator was the number reporting VI, which may not have been the whole sample) (Supplement A1). We produced forest plots of study estimates by recall period, presenting all results from general and higher-risk populations separately. Based on our previous review on AI practices among youth [30], we anticipated substantial heterogeneity across estimates and we therefore pooled results using random-effects models and conducted extensive sub-group analyses to explore the influence of participant and study characteristics and study quality [31–33]. We examined the effect of participant and study characteristics on pooled AI prevalence estimates by conducting sub-group analyses for each recall period; analyses were restricted to recall periods with at least five studies. We examined time trends by dichotomizing at the median survey year of included studies (2005). Measures of study quality and potential sources of bias were also tested using sub-group analyses. Pooled estimates were derived using maximum-likelihood random-effects models based on inverse-variance [34–36] with the procedure “Metafor” [37] in R version 3.2.0. Heterogeneity across study estimates was investigated using I^2^ statistics [38,39].

#### Frequency data

To facilitate comparison across studies, we standardized sex act frequency estimates to one month (Supplement B1). For studies not reporting frequency over this time period, we derived the fraction of sex acts that were AI or UAI and the fraction of UAI and UVI from relevant extracted data when provided (Supplement B2&3). Few studies reported mean frequency of sex acts estimates or 95%CIs; therefore, we were only able to graphically explore the effect of gender, partner type, province, population, interview method and original recall period on (i) the fraction of sex acts that were AI, (ii) the fraction of unprotected sex acts that were UAI through scatter plots.

## Results

### Search results

Supplementary Figure 1 summarizes the study selection procedure and search results. Of the 2520 titles initially identified, 41 articles were included. Most articles were identified from the database search, with three included articles identified through reference scanning and none through the internet search for grey literature. Additional information was obtained from three of the eleven authors contacted. A list of excluded articles is available on request.

### Study characteristics

Table 1 provides a summary of the participant and study characteristics and markers of study quality. Details of each individual study are available in Supplementary Table S1. Of the 41 studies included, 29 and 14 were conducted among general and higher-risk populations, respectively, including two studies which reported on both risk groups separately [40,41]. AI prevalence and AI frequency were reported over various recall periods by 31 (including four studies reporting UAI prevalence only [42–45]) and 14 studies, respectively. No studies reported on lubricant use or condom breakage during AI.

**Table 1.** Summary of study and participant characteristics and study quality of included studies

		General risk *N* = 29 Sources		Higher risk *N* = 14 Sources		Total *N* = 41^b^
**A. Outcomes and key study characteristics**						
Outcomes reported	AI prevalence	21	[40,47–66]	7	[40,67–72]	27^b^
	UAI prevalence only ^a^	4	[42–45]	0	-	4
	AI frequency	6	[41,43,54,73–75]	9	[41,46,70,72,76–80]	14 ^b^
AI prevalence recall period	Lifetime	10	[48,51,52,56–58,60–62,64]	2	[71,72]	12
	12 Months	1	[49]	0	-	1
	6 Months	3	[45,54,63]	0	-	3
	3 Months	6	[42,43,53,59,62,66]	0	-	6
	1 Month	3	[44,47,50]	1	[70]	4
	Current partner	1	[40]	1	[40]	1^b^
	General^c^	2	[55,65]	0	-	2
	Not stated	0	-	3	[67–69]	3
AI frequency recall period	6 Months	1	[54]	0	-	1
	3 Months	3	[41,43,73]	3	[41,77,80]	5^b^
	42 Days	0	-	1	[78]	1
	1 Month	2	[74,75]	3	[70,76,79]	5
	1 Week	0	-	2	[46,72]	2
Reported by partner type	AI prevalence	3	[53–55]	0	-	3
	AI frequency	1	[54]	1	[46]	2
Gender	Male & female	8	[44,45,49,55,56,62,73,74]	0	-	8
	Female only	13	[42,43,47,50–52,59,60,63–66,75]	6	[46,67,68,70–72]	19
	Male only	2	[40,53]	2	[40,69]	3^b^
	Mixed only	6	[41,48,54,57,58,61]	6	[41,76–80]	11^b^
Mean age	<25 years	15	[48–51,55–62,65,66,73]	2	[67,71]	17
	25+ years	13	[40–44,47,52–54,63,64,74,75]	11	[40,41,46,68–70,76–80]	22^b^
	Not stated	1	[45]	1	[72]	2
Study sample	Community	18	[40–45,47–49,51–53,59–61,64,66,73]	0	-	18
	Community and shebeen	1	[74]	0	-	1
	Shebeen	2	[44,75]	0	-	2
	University	2	[40,55]	0	-	2
	School	4	[56–58,62]			4
	Clinic	3	[50,63,65]	0	-	3
	VCT	1	[54]	0	-	1
	STI clinic patients	0	-	4	[40,41,77,79]	4
	HIV-infected	0	-	3	[76,78,80]	3
	FSW	0	-	5	[46,67,68,71,72]	5
	Clients of FSW	0	-	1	[69]	1
	“High risk”^d^	0	-	1	[70]	1
Province	Western Cape	11	[40,41,43,44,48,56,58,62,73–75]	4	[41,77–79]	14^b^
	KwaZulu-Natal	4	[50,52,64,65]	8	[40,46,67–70,72,80]	11^b^
	Elsewhere, multiple or not stated	16	[40,42,45,47,49,51,53–55,57,59–61,63,64,66]	2	[71,76]	18
Urban or rural	Urban	24	[40–45,47,48,50,52–54,56,58–62,64–66,73–75]	11	[40,41,46,70–72,76–80]	33^b^
	Rural	6	[50–52,57,63,65]	0		6
	Mixed or NS	3	[49,55,64]	3	[67–69]	5^b^
Survey year	Pre-2005	12	[40–43,49,54,55,57,58,64,65,73]	9	[40,41,46,67–72]	19^b^
	2005 onwards	17	[44,45,47,48,50–53,56,59–63,66,74,75]	5	[76–80]	22
**B. Study quality and potential for bias**						
Interview method	ACASI	8	[47,48,53,56,60,64,66,75]	3	[76,77,79]	11
	SAQ	9	[41,43,44,48,55,57,58,73,74]	3	[41,68,69]	11^b^
	ACASI or SAQ	1	[62]	0	-	1
	SAQ or FTFI	0		1	[72]	1
	FTFI	13	[40,42,45,47,49–52,54,59,61,63,65]	5	[40,46,70,71,80]	17^b^
	Coital diary	0	-	1	[46]	1
	Telephone	0	-	1	[78]	1
	Not stated	0	-	1	[67]	1
Study design	Cross-sectional	18	[40,41,43,44,48,49,51,54–58,60–63,73,74]	10	[40,41,46,67,69–71,76,78,80]	26^b^
	Cohort	2	[65,75]	2	[68,77]	4
	RCT	9	[42,45,47,50,52,53,59,64,66]	2	[72,79]	11
Sampling method	Convenience	19	[40–44,47,50,51,54,55,59,61,63–66,73–75]	12	[40,41,67–69,71,72,76–80]	29^b^
	SRS	2	[48,49]	0	-	2
	CRS	5	[53,56–58,62]	0	-	5
	RDS	1	[60]	0	-	1
	Not stated	2	[45,52]	2	[46,70]	4
AI first mentioned	Title	2	[41,49]	2	[41,67]	3^b^
	Abstract	5	[43,56–58,62]	4	[46,69,76,79]	9
	Text	22	[40,42,44,45,47,48,50–55,59–61,63–66,73–75]	8	[40,68,70–72,77,78,80]	29^b^
Response rate	≥80%	9	[41,43,44,48,62,65,73–75]	2	[41,69]	10^b^
	<80%	0	-	2	[77,79]	2
	Not stated	20	[40,42,45,47,49–61,63,64,66]	10	[40,46,67,68,70–72,76,78,80]	29^b^
Hetero only^e^	Yes	5	[40,41,53,58,61]	3	[40,41,69]	6^b^
	No	11	[44,45,48,49,54–57,62,73,74]	5	[76–80]	16
	Not applicable^f^	13	[42,43,47,50–52,59,60,63–66,75]	6	[46,67,68,70–72]	19
Language of survey	Regional lang. only	1	[57]	2	[69,78]	3
	Regional lang. & English	17	[40,41,43,44,47–49,53,54,56,59–62,73–75]	7	[40,41,70,76,77,79,80]	22^b^
	Not stated	11	[42,45,50–52,55,58,63–66]	5	[46,67,68,71,72]	16

AI – anal intercourse, VI – vaginal intercourse, UAI – unprotected anal intercourse, UVI – unprotected vaginal intercourse, F – female, M – male, Mix – data available on mixed gender only, FSW – female sex worker, MSM – men who have sex with men, shebeen – an informal establishment serving alcohol, STI – sexually transmitted infection, VCT – voluntary counselling and testing, ACASI – audio computer-assisted self-interview, FTFI – face-to-face interview, SAQ – self-administered questionnaire, CRS – cluster random sample, SRS – simple random sample, RDS – respondent-driven sample, RCT – randomized controlled trial ^a^Studies which reported AI prevalence for unprotected AI only. ^b^Sum lower than expected as two studies report on both higher and general-risk populations. ^c^Recall period referred to here as “general” when participants were asked “Do you practise anal sex”, or similar. ^d^Defined by author as “high risk” but no definition provided; sample consists of 79% FSW [70]. ^e^Whether studies including reported data only on heterosexual anal sex specifically or excluded men who reported having male sexual partners. ^f^Studies sampling women only. It was assumed that the AI reported in these studies was heterosexual only.

Over twice as many studies reported on females than males or mixed gender only. Most studies were conducted among participants with a mean age of twenty-five years or over. The majority of studies on general-risk populations recruited from the community, while the majority on higher-risk populations were of FSW or their clients. Most studies were conducted in the Western Cape or KwaZulu-Natal, with the large majority recruiting in urban settings.

### Study quality and potential bias

Sample size tended to be larger in studies reporting on general risk as opposed to higher-risk groups (Supplementary Table 1). The most commonly used interview method was face-to-face interview (FTFI), followed by audio computer-assisted self-interview (ACASI) and self-administered questionnaire (SAQ) (Table 1B). Three studies directly compared reports of AI practice using different methods [46–48]. The majority of studies were cross-sectional and used convenience sampling with only one nationally representative survey [49]. Most studies first mentioned AI in the main text. Response rate was not reported by most studies. Although we excluded studies that explicitly stated that the sample included MSM, only six studies among men or mixed gender reported asking about heterosexual AI specifically or excluded men who reported having male partners. Half of the studies reporting AI frequency data did not report the proportion of the sample that were sexually active (data not shown).

### How common is AI?

**Figure 1.**
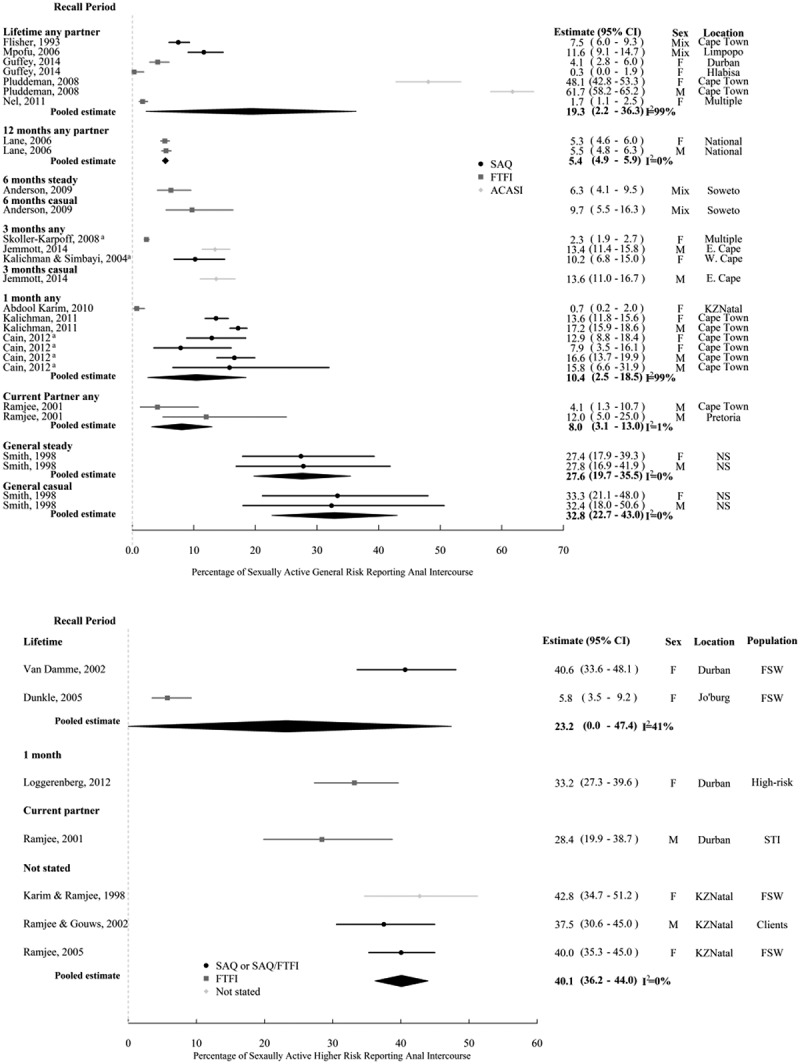
Prevalence of AI over the various recall periods reported. Study estimates of AI prevalence among heterosexual men and women among (a) general-risk study participants and (b) higher-risk study participants. Shown on the graph, study estimates are ordered by survey year and 95% confidence intervals (95% CI) and Higgins I^2^ [29]. I^2^ can lie between 0 and 100%; where 0% and 100% indicate no and the most observed heterogeneity across study estimates. ^a^AI prevalence with steady partners has been grouped with any partner type here. ^b^Estimates are for unprotected AI only. ^C^12 of the 18 school classes recruited used ACASI, the remainder used SAQ. ^d^Recruited from shebeens. ^e^Recruited from the community. ACASI = audio computer-assisted self-interview, FTFI = face-to-face interview, SAQ = self-administered questionnaire; F = female, M = male, Mix = data available for mixed gender only; Clients = clients of female sex workers, FSW = female sex workers, High risk = defined by authors as being at high risk of HIV infection (79% were FSW), STI = STI clinic patients.

Figures 1a and b show independent AI prevalence study estimates among sexually active respondents, for general and key populations at higher risk, respectively. AI prevalence estimates in general-risk populations ranged from 0.4% to 70.0% across recall periods. Estimates over the same recall period coming from different studies were very heterogeneous (I^2^ ≥ 90%). The two highest AI prevalence estimates were reported by male school pupils (61.7% and 70.0%) in studies using ACASI [46] and a mixture of ACASI and SAQ [49]. In contrast, the lowest estimates (≤3% over various recall periods) were reported by adult women in FTFI [42,47,50–52,59,65](Figure 1a). Apart from one estimate [81], AI prevalence among higher-risk respondents was consistently high across recall periods (28.4–42.8%) (Figure 1b) and generally higher than for general-risk populations.

### Who practises AI the most?

#### Study and participant characteristics

Figure 2a displays pooled estimates from sub-group analyses of AI prevalence for the recall periods (lifetime and three months) and risk populations (general-risk populations only) with sufficient study estimates. The only clear statistical difference in pooled estimates were between those from urban and rural samples, with urban estimates being higher (lifetime prevalence: 21.1% (95%CI 10.2–32.0%, *N* = 8) vs. 4.5% (95%CI 0.0–10.1%, *N* = 3)). Although CIs of pooled estimates by sub-group of other variables overlapped in either one or both recall periods, the magnitude of the pooled estimates was larger for males than females (e.g. lifetime prevalence: 48.4% (95%CI 30.0–66.8%, *N* = 2 vs. 14.5% (95%CI 4.5–24.5%, *N* = 6)) and in samples recruited from schools compared to communities. Pooled estimates were also higher in samples with mean age below twenty-five years and in studies conducted after 2005. Although pooled estimates are higher in the Western Cape compared to elsewhere, this is likely confounded by interview methods.

**Figure 2.**
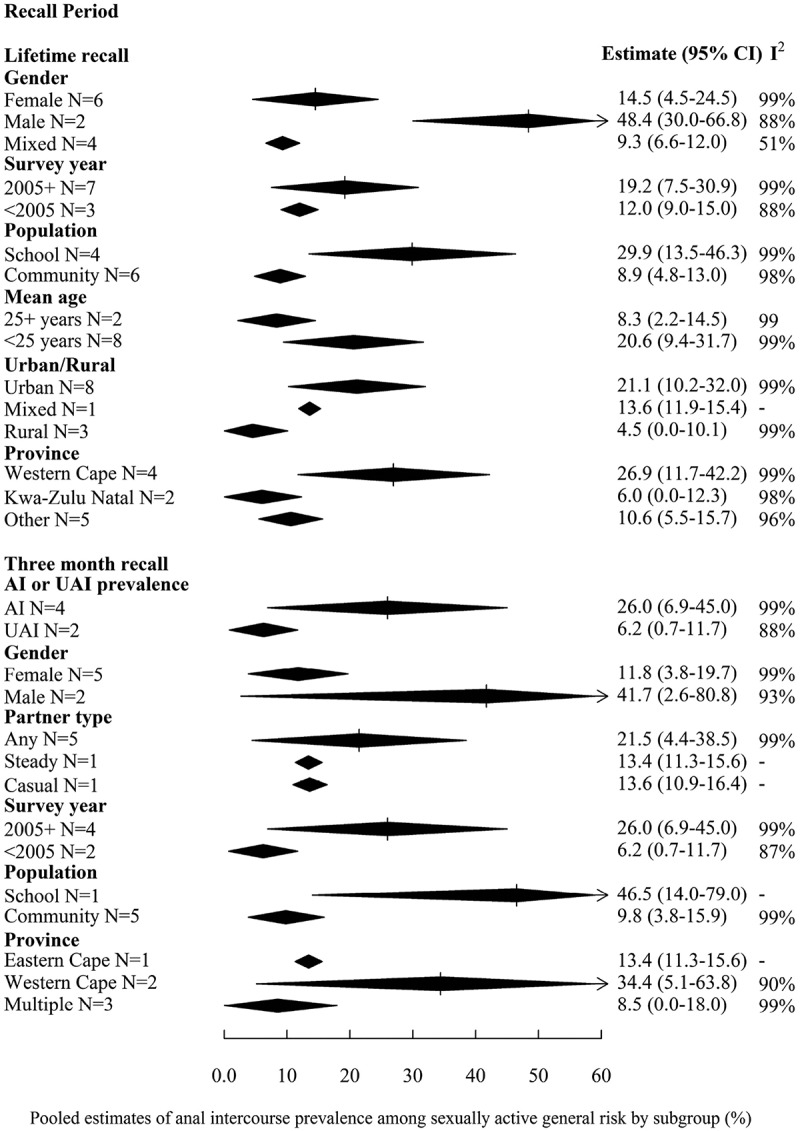
Forest plot of sub-group analyses of prevalence of AI among sexually active general-risk populations; study and participant characteristics. Results are presented for recall periods reported by at least five studies (lifetime and three months) on (A) study and participant characteristics and (B) study quality.I^2^ is calculated as described in Higgins et al. [39]. I^2^ lies between 0 and 100 %; 0 % indicates no observed heterogeneity and larger values show increasing heterogeneity. AI = anal intercourse, UAI = unprotected anal intercourse, ACASI = audio computer-assisted self-interview, FTFI = face-to-face interview, SAQ = self-administered questionnaire, CRS = cluster random sampling, RCT = randomized control trial, NS = not stated. Shebeens are informal alcohol serving establishments. One study reported prevalence for casual and steady partners over three month recall. Prevalence for steady partners only was pooled from this study, except when comparing prevalence by partner type. Mean age was not examined in three month recall as all studies recruiting from the community either did not report on mean age, or had a mean age of 25+ years. Sub-group analysis of population (school vs. community) acts as proxy for analysis by age. Neither of the studies reporting on past three months explicitly included heterosexuals only.

#### Study quality and potential for biases

Figure 2b presents the subgroup analyses assessing the influence of study quality among general-risk populations. The only measure of study quality that clearly influenced AI estimates was interview method, while CIs overlapped for other variables. Pooled estimates were lower for studies using FTFI compared to ACASI or SAQ over both recall periods, with estimates highest for ACASI in lifetime, but not past three months’ recall. For example, pooled lifetime AI prevalence was 3.2% (95%CI 0.9–5.4%, *N* = 3), 8.4% (95%CI 5.5–11.2%, *N* = 3) and 28.5% (95%CI 13.2–43.9%, *N* = 4) using FTFI, SAQ and ACASI, respectively. Pooled estimates of convenience samples were lower than for other sampling methods, while those from cross-sectional studies were higher than from other study types. Studies not explicitly stating that the sample was heterosexual only, and those that stated using English and regional languages compared to not stating language used tended to have higher estimates. Pooled estimates were higher when AI was first mentioned in the abstract compared to the main text. The sole study which mentioned AI in the title reported the highest AI prevalence [67].

**Figure 2.**
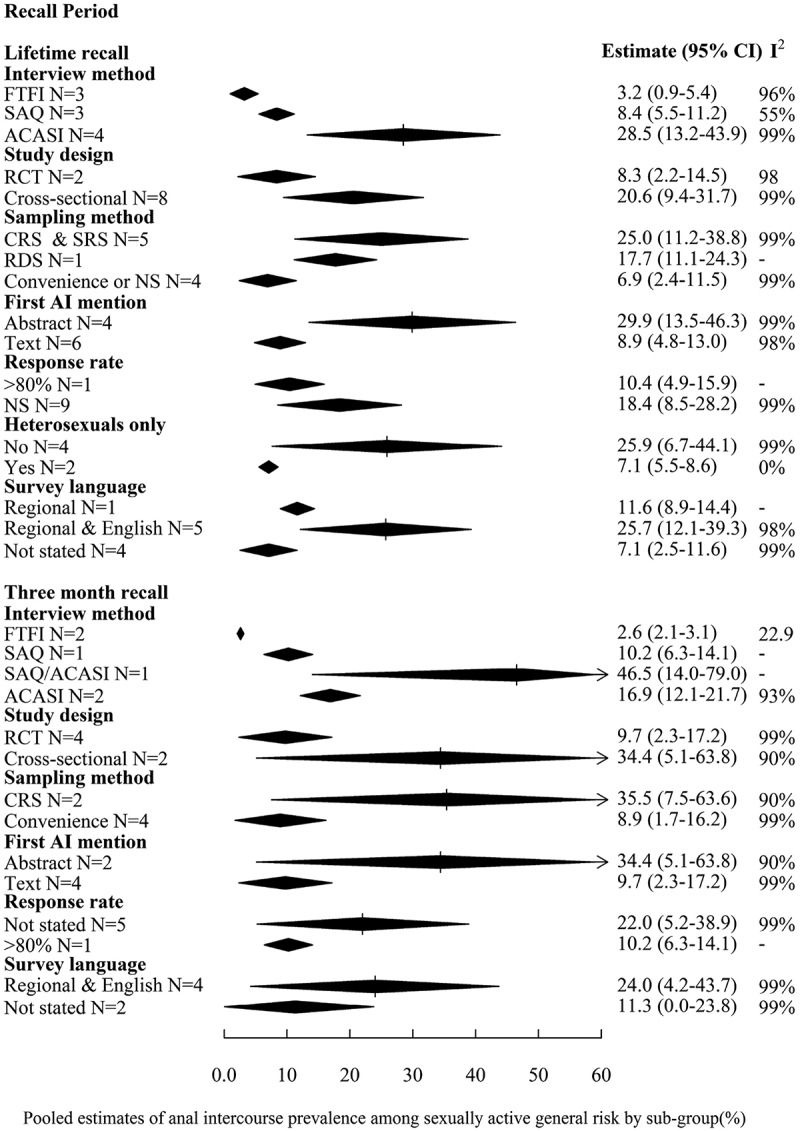
(Continued)

We were unable to conduct sub-group analyses by participant or study characteristics or study quality for higher-risk populations given the few estimates per recall period (Figure 1b). Only two studies reported on higher-risk males compared to five on females, all of which sampled FSW, or predominantly FSW, with no discernible difference in prevalence by gender or population (Figure 1b). No studies on higher-risk populations reported prevalence by partner type and all were conducted before 2005 (Figure 1b) and in urban settings (Table 1). All but one AI prevalence estimates were from KwaZulu-Natal, with the lowest prevalence across recall periods reported in the sole study from Gauteng [71]. All studies on higher-risk respondents used convenience samples or failed to specify sampling method [82], and response rate was only reported by one study [69]. Both studies on males explicitly stated that they consisted of heterosexuals only [40,69].

### How frequent is AI among heterosexuals?

**Figure 3.**
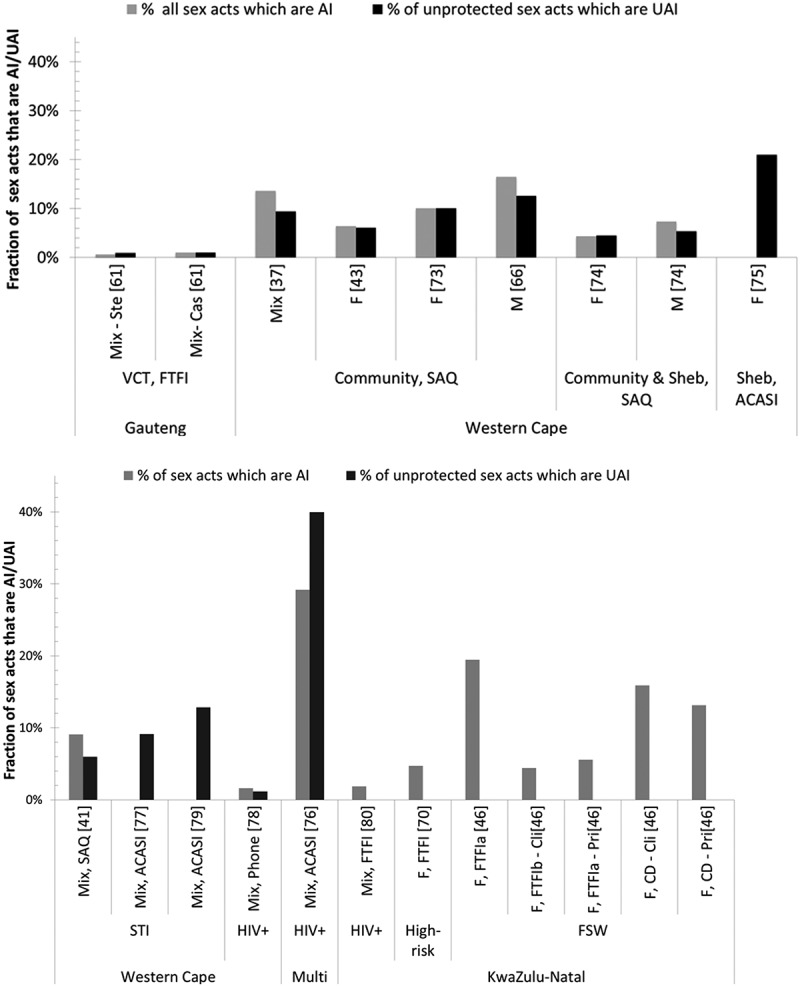
Bar chart of fraction of sex acts that are AI and fraction of unprotected acts that are UAI. Among (A) general-risk study participants and (B) higher-risk study participants. AI = anal intercourse, VI = vaginal intercourse, UAI = unprotected anal intercourse, ACASI = audio computer-assisted self-interview, FTFI = face-to-face interview, SAQ = self-administered questionnaire, F = female, M = male, Mix = data available for mixed gender only, N = sample size, FSW = female sex workers, High risk = defined by authors as high risk of HIV infection, 79% were FSW, STI clinic = sexually transmitted infections clinic patients, VCT = voluntary counselling and testing,. Cli = with clients, Cas = with casual partners, Pri = with primary partner, Ste = with steady partner. Sheb = Shebeen, which are informal drinking establishments. All studies with available data were included.

The mean number of AI acts per month across whole samples (i.e. including those reporting no AI) tended to be higher among higher-risk populations (AI frequency: 0.1–16.9, *N* = 9; UAI frequency: 0.1–0.5, *N* = 5) than general-risk populations (AI frequency: 0.1–1.1, *N* = 7; UAI frequency: 0.01–0.7 *N* = 7; Figure S2a&b and Table S2 in Supplementary file). Thus, the fraction of all sex acts that were AI or UAI was slightly higher for higher-risk than general-risk populations. Among higher risk, 0.6–29.2% acts (*N* = 6) were AI and 1.2–40.0% (*N* = 5) were UAI (Figure 3b), compared with 0.6–16.5% acts (*N* = 6) and 0.7–21.0% (*N* = 7) for AI and UAI, respectively, among general-risk populations (Figure 3a). Condom use during AI was similar to that for VI. Among general-risk populations, the fraction of AI and VI acts that were unprotected was 27.0–53.6% and 26.9–57.0%, respectively (*N* = 5) (Figure S2a and Table S2 in Supplementary file).

*Who practises AI most frequently?* General-risk males reported a higher number of AI acts and tended to report a slightly larger fraction of AI but a similar fraction of UAI compared to women (Table S2 & Figure S3a in Supplementary file). The fraction of sex acts that were AI was similar across partner types in both risk groups (Figure S3a&b). A smaller fraction of sex acts were AI in samples recruited from VCT services than from communities or shebeens among the general-risk population but estimates among higher-risk groups did not vary by population.

#### Potential sources of bias

As with AI prevalence, confidentiality of interview method seemed to affect reporting of AI frequency among general-risk populations, with the lowest fraction of sex acts that were AI being found in the only study using FTFI [61] and the highest fraction of UAI found in the only study using ACASI [75] (Figures S3a&3a). Studies among higher-risk populations used a wider variety of interview methods and differences between methods were less clear than among general-risk populations (Figures S3b), although both the highest fraction of AI and UAI were reported using ACASI (Figures 3b&S3b). A FSW study comparing FTFI and pictorial coital diaries [46] documented a substantially higher fraction of AI sex acts through coital diary than daily FTFI (Table S2, Figures 3b&S3b in Supplementary file). Greater numbers of both types of sex acts were reported over shorter recall periods (when standardized to one month) (Table S2 in Supplementary file).

## Discussion

Our review adds substantially to the current literature and understanding of AI practices in South Africa. Our findings suggest that heterosexual AI is commonly practised by both men and women in South Africa. Both AI prevalence and frequency tended to be higher among higher-risk populations. Among general-risk populations, reported AI prevalence tended to be higher when more confidential interview methods were used, in urban areas, as well as among males and younger people, particularly adolescents. This latter finding is particularly concerning given adolescent girls’ increased vulnerability to HIV infection [19].

Previous modelling studies suggest that only 5–10% of unprotected sex acts being UAI could explain a substantial fraction of HIV infections among women [8,12]. As such, the frequency of UAI found in this review (0.7–21.0% of unprotected acts being UAI among whole samples of general-risk populations) implies that heterosexual AI may be a significant driver of South Africa’s HIV epidemic. The reported fractions of sex acts that are AI are high given that the majority of participants in all studies reported not practicing AI, implying that those who do practise AI, practise it frequently. For example, in the two studies of general-risk participants which reported AI frequency solely among those reporting AI, between 8.9% and 43.2% of all sex acts were AI [43,54]. Although qualitative research in South Africa has found low awareness of HIV transmission risk during AI acts [9–11], our review suggests that contrary to expectation, condoms tended to be used as or slightly more often during AI than VI acts in both risk groups.

AI prevalence did not increase with longer recall periods as would be expected if people do not practice AI all their life; instead, recall period had no discernible effect, which we also found in our systematic review on AI practice among young people [5]. These observations suggest that either reporting accuracy decreases over time, as previously reported in other sexual behaviour studies [83–85], and/or that those who initiate AI continue to practice it all their life. Unfortunately, only one included study reported AI prevalence over a short and a long recall period which prevented within-study comparisons [62]. Reporting AI over both short and longer recall periods is desirable because it provides an indication of reporting accuracy by recall period (e.g. if AI is lower over longer recall period within the same sample) and also indirectly provides information on how long people practise AI for (e.g. if AI is higher over longer recall period). Most studies reported estimates of AI prevalence over a lifetime only. While this information is necessary, it is insufficient to fully reflect the current level of HIV risk due to AI in the population since many may have ceased practicing AI.

Moreover, many studies only report AI prevalence but did not provide any information on AI frequency compared to VI among those who report AI, which again limits our ability to estimate the contribution of AI to overall HIV transmission at the population level. Indeed, only a third of the studies included in this review (14/41) reported information on AI frequency with only half of those reporting data for the four types of sexual contact (AI, VI, UAI, UVI). Having information on AI and VI frequency is crucial to estimating the contribution of AI on annual HIV incidence in a population [86]. Of the nine studies reporting frequency which included males, only two reported results by gender [73,74], two reported by partner type [46,54] and none by age group. Among both risk groups, the number of reported sex acts (both VI and AI) unexpectedly decreased as recall periods increased, confirming that sex act data is more accurately remembered over shorter periods such as one month [84].

AI is often a highly stigmatized behaviour [9,10] leading to social desirability bias. Therefore, it may be more willingly reported using more confidential interviewing methods [5,87,88]. Our sub-group analyses found that reported AI prevalence increased with increasing confidentiality of interview method, a finding mirrored in our previous review on young people [5]. The highest number of AI acts was recorded among FSW completing daily pictorial coital diaries, with the same study finding a substantially lower number reported through daily FTFI and lower again through weekly FTFI [46]. Likewise, the few studies using confidential ACASI found the highest fraction of AI and UAI acts among both general- and higher-risk populations. Together, these findings support the need to use more confidential methods in the reporting of AI practices, but also highlight the importance of using short recall periods to record frequency data. AI may be more stigmatized for women than men, so the lower reported prevalence among women may partly be explained by greater social-desirability bias in women reporting stigmatized sexual behaviour [89]. This difference may also reflect greater fractions of men buying sex than women selling sex; thus more men than women are likely to engage in high risk behaviour, including AI.

All but one of the included RCTs testing vaginal microbicides or vaginal rings used FTFI and reported low AI prevalence (≤3% across recall periods) [42,50,51,59]. The VOICE microbicide trial, however, used ACASI and found AI prevalence to be over six times higher than other trials [66]. This suggests that in order to understand to what extent AI practice may be interfering with the efficacy of vaginal tract interventions, FTFI should be avoided. Qualitative work exploring participants’ understanding of questions on AI found that they were often misunderstood; in particular, AI was often confused with other sexual practices such as vaginal sex “from behind” [24,25]. Therefore, although more confidential methods reduce social desirability bias, and thus often elicit higher responses on sensitive questions, they also have the drawback of offering little or no opportunity for clarification of misunderstood questions [24]. Reporting may be improved by using clearer questions on AI and visual aids, such as the study using unambiguous pictorial coital diaries which found the highest number of AI acts reported in this review [46].

This review has a number of limitations. We did not include studies published earlier than 1990, as we were most interested in behaviour in the context of South Africa’s HIV epidemic, which started to explode in the 90s. Our use of engagement in VI as the definition of sexual activity may mask the practice of AI by those who do not engage in VI and for shorter recall periods this definition may selectively include individuals with higher sexual activity. We compared AI prevalence across samples with varying levels of sexual activity; this may have introduced bias as samples among young adolescents (mean age <16 years) as those who are sexually active at young ages may have higher-risk behaviour. All included papers referred either to “anal sex” or “anal intercourse”, which may be ambiguous terms that could include non-penetrative sexual activity; our assumption that this refers only to penile-anal intercourse may have inflated estimates in this review. We included studies on men that did not explicitly state that MSM were excluded from the sample, and thus study estimates may include some homosexual AI. As the majority of studies employed convenience sampling, we cannot be confident that the included studies are representative of the various populations.

The largest limitation to our analysis is the wide variability of reporting methods both for prevalence and frequency across studies. Different recall periods hindered comparison of AI prevalence across studies and limited interpretation of sub-group analyses. Frequency was also reported in a variety of ways, with only a fifth of studies with frequency data reporting CI or SD on all types of sex acts [41,76,90], which prevented us from pooling or conducting detailed subgroup analyses of frequency data. Finally, no included study reported on condom breakage nor whether, or what type of lubricant was used during AI. Condoms break more frequently during AI than VI [91–93], which may have affected the accuracy of UAI estimates. Understanding lubricant use is important as the use of water-based lubricant may reduce the likelihood of condom breakage [94] while oil-based lubricant, which is commonly used in sub-Saharan Africa, may increase the likelihood of breakage by degrading the latex [11,94].

Our review was greatly strengthened by using wide search terms, for example, omitting the word “anal”, ensuring we captured studies for which AI practice was not a primary outcome variable. Given that both prevalence and frequency tended to be lower the later in the article that AI was first mentioned, we limited the impact of publication bias, thus increasing the accuracy of our results.

This review provides valuable information that can be used to guide policy, research and survey design both within South Africa and internationally as well as to inform future mathematical models of South Africa’s HIV epidemics. AI is widely practised by diverse South African heterosexual populations. Given its high risk of HIV transmission, questions on its practice should be routinely included in surveys on sexual behaviour, particularly in routine national surveys so that trends over time can be examined. Accuracy of AI estimates can be improved by using visual aids, ideally combined with confidential interview methods in order to reduce social-desirability bias. In order to obtain more epidemiologically useful estimates, surveys should report AI prevalence over lifetime and shorter recall periods such as past three months. Frequency data on number of both protected and unprotected AI and VI acts and confidence intervals should be reported over one month and one week. Such data could powerfully inform the extent to which AI impacts on South Africa’s HIV epidemic.

## Key messages

- Heterosexual anal intercourse is common across population groups in South Africa and thus may be contributing substantially to the country’s HIV epidemic.
- The prevalence of anal intercourse is usually higher in key populations at higher risk; prevalence among sexually active general-risk samples tended to be higher in urban areas, among males, and among younger people, particularly adolescents.
- Condoms tended to be used as often during anal intercourse as vaginal intercourse.
- Higher prevalence and frequency of anal intercourse tended to be reported when using more confidential interview methods
- In order to better understand the extent to which heterosexual anal intercourse contributes to South Africa’s HIV epidemic, standardized recall periods and confidential interview methods should be used in future studies of this stigmatized behaviour.

## Notes

To access the supplementary material to this article please see Supplementary Files under Article Tools online.

## References

[1] Brody S, Potterat JJ. Assessing the role of anal intercourse in the epidemiology of AIDS in Africa. Int J STD AIDS. 2003 7;14(7):431–436. DOI:10.1258/095646203765371330 12869220

[2] Mâsse BR, Boily M-C, Dimitrov D, Desai K Efficacy dilution in randomized placebo-controlled vaginal microbicide trials. Emerg Themes Epidemiol. 2009 1;6:5 DOI:10.1186/1742-7622-6-5 19818138 PMC2768687

[3] Baggaley RF, White RG, Boily M-C HIV transmission risk through anal intercourse: systematic review, meta-analysis and implications for HIV prevention. Int J Epidemiol. 2010 8;39(4):1048–1063. DOI:10.1093/ije/dyq057 20406794 PMC2929353

[4] McGowan I, Taylor DJ Heterosexual anal intercourse has the potential to cause a significant loss of power in vaginal microbicide effectiveness studies. Sex Transm Dis. 2010 6;37(6):361–364. DOI:10.1097/OLQ.0b013e3181bf55a0 20514687

[5] Owen BN, Brock PM, Butler AR, Pickles M, Brisson M, Baggaley RF, et al Prevalence and frequency of heterosexual anal intercourse among young people: a systematic review and meta-analysis. AIDS Behav. 2015 1 25 DOI:10.1007/s10461-015-0997-y 25618257

[6] Baggaley RF, Dimitrov D, Owen BN, Pickles M, Butler AR, Masse B, et al Heterosexual anal intercourse: a neglected risk factor for HIV?. Am J Reprod Immunol. 2013 2;69(Suppl 1):95–105. DOI:10.1111/aji.12042 23279040 PMC3938911

[7] Fox J, White PJ, Weber J, Garnett GP, Ward H, Fidler S Quantifying sexual exposure to HIV within an HIV-serodiscordant relationship: development of an algorithm. Aids. 2011 5 15;25(8):1065–1082. 21537113 10.1097/QAD.0b013e328344fe4a

[8] Boily MC The relative contribution of anal intercourse and primary infection to mature heterosexual HIV epidemics. Sex Transm Infect. 2011 7 10;87(Suppl 1):Abstract 01–S07.01.

[9] Makhubele MB, Parker W Heterosexual anal sex amongst young adults in South Africa: risks and perspectives. Johannesburg. 2014 Available from: http://cadre.org.za/publications-and-presentations/

[10] Stadler JJ, Delany S, Mntambo M Sexual coercion and sexual desire: ambivalent meanings of heterosexual anal sex in Soweto, South Africa. AIDS Care. 2007 11;19(10):1189–1193. DOI:10.1080/09540120701408134 18071961

[11] Duby Z, Hartmann M, Montgomery ET, Colvin CJ, Mensch B, van der Straten A Condoms, lubricants and rectal cleansing: practices associated with heterosexual penile-anal intercourse amongst participants in an HIV prevention trial in South Africa, Uganda and Zimbabwe. AIDS Behav. 2015 7 1;4:754–762. 10.1007/s10461-015-1120-0 PMC4698090 26126586

[12] Boily M-C, Dimitrov D, Abdool Karim SS, Mâsse B The future role of rectal and vaginal microbicides to prevent HIV infection in heterosexual populations: implications for product development and prevention. Sex Transm Infect. 2011 12;87(7):646–653. DOI:10.1136/sextrans-2011-050184 22110117 PMC3332062

[13] Louissaint NA, Cao Y-J, Skipper PL, Liberman RG, Tannenbaum SR, Nimmagadda S, et al Single dose pharmacokinetics of oral tenofovir in plasma, peripheral blood mononuclear cells, colonic tissue, and vaginal tissue. AIDS Res Hum Retroviruses. 2013 11;29(3):1443–1450. DOI:10.1089/aid.2012.0059 23600365 PMC3809387

[14] Patterson KB, Prince HA, Kraft E, Jenkins AJ, Shaheen NJ, Rooney JF, et al Penetration of tenofovir and emtricitabine in mucosal tissues: implications for prevention of HIV-1 transmission. Sci Transl Med. 2011 12 7;3(112):112re4. 10.1126/scitranslmed.3003174 PMC3483088 22158861

[15] Grant RM, Lama JR, Anderson PL, McMahan V, Liu AY, Vargas L, et al Preexposure chemoprophylaxis for HIV prevention in men who have sex with men. N Engl J Med. 2010 12 30;363(27):2587–2599. 21091279 10.1056/NEJMoa1011205 PMC3079639

[16] Marrazzo JM, Ramjee G, Richardson BA, Gomez K, Mgodi N, Nair G, et al Tenofovir-based preexposure prophylaxis for HIV infection among African Women. N Engl J Med. 2015 2 5;372(6):509–518. 25651245 10.1056/NEJMoa1402269 PMC4341965

[17] Hendrix CW, Chen BA, Guddera V, Hoesley C, Justman J, Nakabiito C, et al MTN-001: randomized pharmacokinetic cross-over study comparing tenofovir vaginal gel and oral tablets in vaginal tissue and other compartments. Plos One. 2013 1;8(1):e55013 DOI:10.1371/journal.pone.0055013 23383037 PMC3559346

[18] Republic of South Africa Global AIDS Response Progress Report 2012. Pretoria; 2012.

[19] Dellar RC, Dlamini S, Karim QA Adolescent girls and young women: key populations for HIV epidemic control. J Int AIDS Soc. 2015 1;18(2 Suppl 1):19408 DOI:10.7448/IAS.18.2.19408 25724504 PMC4344544

[20] Pettifor A, MacPhail C, Rees H, Cohen M HIV and sexual behavior among young people: the South African Paradox. Sex Transm Dis. 2008 10;35(10):843–844. DOI:10.1097/OLQ.0b013e31817bbcb4 18716569

[21] Gross M, Holte SE, Marmor M, Mwatha A, Koblin BA, Mayer KH Anal sex among HIV-seronegative women at high risk of HIV exposure. The HIVNET Vaccine preparedness study 2 protocol team. J Acquir Immune Defic Syndr. 2000 8 1;24(4):393–398. 11015157 10.1097/00126334-200008010-00015

[22] Veldhuijzen NJ, Ingabire C, Luchters S, Wilkister Bosire SB, Matthew Chersich JVDW Anal intercourse among female sex workers in East Africa is associated with other high-risk behaviours for HIV. Sex Health. 2011;8:251–254. DOI:10.1071/SH10144 21592442

[23] Smith LB, Adler NE, Tschann JM Underreporting sensitive behaviors: the case of young women’s willingness to report abortion. Health Psychol. 1999 1;18(1):37–43. DOI:10.1037/0278-6133.18.1.37 9925044

[24] Duby Z, Hartmann M, Mahaka I, Munaiwa O, Nabukeera J, Vilakazi N, et al Lost in translation: language, terminology, and understanding of Penile-Anal intercourse in an HIV prevention trial in South Africa, Uganda, and Zimbabwe. J Sex Res. 2015;53(9):1096–1106. 10.1080/00224499.2015.1069784 PMC4961617 26566583

[25] Ndinda C, Chimbwete C, McGrath N, Pool R Perceptions of anal sex in rural South Africa. Cult Health Sex. 2008 2;10(2):205–212. DOI:10.1080/13691050600988416 18247212

[26] Mavhu W, Langhaug L, Manyonga B, Power R, Cowan F What is “sex” exactly? Using cognitive interviewing to improve the validity of sexual behaviour reporting among young people in rural Zimbabwe. Cult Health Sex. 2008 8;10(6):563–572. DOI:10.1080/13691050801948102 18649195

[27] Baggaley RF, Dimitrov D, Owen BN, Pickles M, Butler AR, Masse B, et al Heterosexual anal intercourse: a neglected risk factor for HIV?. Am J Reprod Immunol. 2013;69(SUPPL.1):95–105. DOI:10.1111/aji.12042 23279040 PMC3938911

[28] Stroup DF, Berlin JA, Morton SC, Olkin I, Williamson GD, Rennie D, et al Meta-analysis of observational studies in epidemiology: a proposal for reporting. Meta-analysis Of Observational Studies in Epidemiology (MOOSE) group. Jama. 2000 4 19;283(15):2008–2012. 10789670 10.1001/jama.283.15.2008

[29] Moher D, Liberati A, Tetzlaff J, Altman DG The PRISMA group preferred reporting items for systematic reviews and meta-analyses: the PRISMA statement. Plos Med. Public Library of Science. 2009 7 21;6(7):e1000097. DOI:10.1371/journal.pmed.1000097 PMC2707599 19621072

[30] Owen BN, Brock PM, Butler AR, Pickles M, Brisson M, Baggaley RF, et al Prevalence and frequency of heterosexual anal intercourse among young people: a systematic review and meta-analysis. AIDS Behav. 2015;19(7):1338–1360. DOI:10.1007/s10461-014-0887-8 25618257

[31] Borenstein M, Higgins JPT Meta-analysis and subgroups. Prev Sci. 2013 4;14(2):134–143. DOI:10.1007/s11121-013-0377-7 23479191

[32] Higgens JPT, Green S Cochrane handbook for systematic reviews of interventions. 5.1.0 ed. The Cochrane Collaboration; 2011 Available from: www.handbook.cochrane.org.

[33] Ioannidis JPA, Patsopoulos NA, Rothstein HR Reasons or excuses for avoiding meta-analysis in forest plots. BMJ. 2008 6 21;336(7658):1413–1415. 18566080 10.1136/bmj.a117 PMC2432114

[34] Egger M, Smith GD, Altman DG, editors. Systematic reviews in health care: meta-analysis in context Second. London: Wiley; 2008.

[35] Van Houwelingen HC, Arends LR, Stijnen T Advanced methods in meta-analysis: multivariate approach and meta-regression. Stat Med. 2002 2 28;21(4):589–624. 11836738 10.1002/sim.1040

[36] Hedges LV, Olkin I Statistical methods for meta-analysis. Orlando, FL: Academic Press; 1985 Available from: https://www.researchgate.net/publication/216811655_Statistical_Methods_in_Meta-Analysis

[37] Viechtbauer W, Viechtbauer W Conducting meta-analyses in R with the metafor package. J Stat Softw. 2010;36(3):1–48. J Stat Softw. 2010;36(3):1–48 DOI:10.18637/jss.v036.i03

[38] Huedo-Medina TB, Sánchez-Meca J, Marín-Martínez F, Botella J Assessing heterogeneity in meta-analysis: Q statistic or I2 index?. Psychol Methods. 2006 6;11(2):193–206. DOI:10.1037/1082-989X.11.2.193 16784338

[39] Higgins JPT, Thompson SG, Deeks JJ, Altman DG Measuring inconsistency in meta-analyses. BMJ. 2003 9 6;327(7414):557–560. 12958120 10.1136/bmj.327.7414.557 PMC192859

[40] Ramjee BG, Gouws E, Andrews A, Myer L, Weber AE The acceptability of a vaginal microbicide among South African men. Int Fam Plan Perspect. 2001;27(4):164–170. DOI:10.2307/2673851

[41] Kalichman SC, Simbayi LC, Cain D, Jooste S Heterosexual anal intercourse among community and clinical settings in Cape Town, South Africa. Sex Transm Infect. 2009;85:411–415. DOI:10.1136/sti.2008.035444 19429569 PMC3017216

[42] Skoler-Karpoff S, Ramjee G, Ahmed K, Altini L, Plagianos MG, Friedland B, et al Efficacy of Carraguard for prevention of HIV infection in women in South Africa: a randomised, double-blind, placebo-controlled trial. Lancet. 2008 12 6;372(9654):1977–1987. 19059048 10.1016/S0140-6736(08)61842-5

[43] Kalichman SC, Simbayi LC Sexual assault history and risks for sexually transmitted infections among women in an African township in Cape Town, South Africa. AIDS Care. 2004 8;16(6):681–689. DOI:10.1080/09540120410331269530 15370057

[44] Cain D, Pare V, Kalichman SC, Harel O, Mthembu J, Carey MP, et al HIV risks associated with patronizing alcohol serving establishments in South African Townships, Cape Town. Prev Sci. 2012 12;13(6):627–634. DOI:10.1007/s11121-011-0241-6 22992872 PMC4540371

[45] Gray GE, Metch B, Churchyard G, Mlisana K, Nchabeleng M, Allen M, et al Does participation in an HIV vaccine efficacy trial affect risk behaviour in South Africa?. Vaccine. 2013 4 12;31(16):2089–2096. 23370155 10.1016/j.vaccine.2013.01.031 PMC3942784

[46] Ramjee G, Weber A, Morar N Recording sexual behavior: comparison of recall questionnaires with a coital diary. Sex Transm Dis. 1999;26(7):374–380. DOI:10.1097/00007435-199908000-00002 10458629

[47] Mensch BS, Hewett PC, Abbott S, Rankin J, Littlefield S, Ahmed K, et al Assessing the reporting of adherence and sexual activity in a simulated microbicide trial in South Africa: an interview mode experiment using a placebo gel. AIDS Behav. 2011 2;15(2):407–421. DOI:10.1007/s10461-010-9791-z 20886278

[48] Jaspan HB, Flisher AJ, Myer L, Mathews C, Seebregts C, Berwick JR, et al Brief report : methods for collecting sexual behaviour information from South African adolescents — a comparison of paper versus personal digital assistant questionnaires. J Adolesence. 2007;30(2):353–359. DOI:10.1016/j.adolescence.2006.11.002 17187853

[49] Lane T, Pettifor A, Pascor S, Fiamma ARH Heterosexual anal intercourse increases risk of HIV infection among young South African men. Aids. 2006;20:112–132. DOI:10.1097/01.aids.0000198083.55078.02 16327330

[50] Abdool Karim Q, Abdool Karim SS, Frohlich JA, Grobler AC, Baxter C, Mansoor LE, et al Effectiveness and safety of tenofovir gel, an antiretroviral microbicide, for the prevention of HIV infection in women. Sci (80-). 2010 9 3;329(5996):1168–1174. 10.1126/science.1193748 PMC3001187 20643915

[51] Nel A, Louw C, Hellstrom E, Braunstein SL, Treadwell I HIV prevalence and incidence among sexually active females in two districts of South Africa to determine microbicide trial feasibility. Plos One. 2011;6(8):6–13. DOI:10.1371/journal.pone.0021528 PMC3154187 21853020

[52] Guffey MB, Richardson B, Husnik M, Makanani B, Chilongozi D, Yu E, et al HPTN 035 phase II/IIb randomised safety and effectiveness study of the vaginal microbicides BufferGel and 0.5% PRO 2000 for the prevention of sexually transmitted infections in women. Sex Transm Infect. 2014 8;90(5):363–369. DOI:10.1136/sextrans-2014-051537 24898857 PMC4278566

[53] Jemmott JB, Jemmott LS, O’Leary A, Ngwane Z, Icard LD, Heeren GA, et al Cluster-randomized controlled trial of an HIV/sexually transmitted infection risk-reduction intervention for South African men. Am J Public Health. 2014 3;104(3):467–473. DOI:10.2105/AJPH.2014.302167 24432923 PMC3953794

[54] Andersson KM, Van Niekerk RM, Niccolai LM, Mlungwana ON, Holdsworth IM, Bogoshi M, et al Sexual risk behaviour of the first cohort undergoing screening for enrollment into Phase I/II HIV vaccine trials in South Africa. Int J STD AIDS. 2009 2;20(2):95–101. DOI:10.1258/ijsa.2008.008207 19182054 PMC3556817

[55] Smith AM, de Visser R, Akande A, Rosenthal D, Moore S Australian and South African undergraduates’ HIV-related knowledge, attitudes, and behaviors. Arch Sex Behav. 1998 6;27(3):279–294. DOI:10.1023/A:1018603102295 9604117

[56] Pluddemann A, Flisher AJ, Mathews C, Plu A, Carney T, Lombard C Adolescent methamphetamine use and sexual risk behaviour in secondary school students in Cape Town, South Africa. Drug Alcohol Rev. 2008;27(6):687–692. DOI:10.1080/09595230802245253 18825548

[57] Mpofu E, Flisher AJ, Bility K, Onya H, Lombard C Sexual partners in a rural South African Setting. AIDS Behav. 2006;10(4):399–404. DOI:10.1007/s10461-005-9037-7 16402280

[58] Flisher A, Ziervogel C Risk-taking behaviour of Cape Peninsula high school students. South Afr. 1993;83(July):495–498. 8211488

[59] Palanee-Phillips T, Schwartz K, Brown ER, Govender V, Mgodi N, Kiweewa FM, et al Characteristics of women enrolled into a randomized clinical trial of dapivirine vaginal ring for HIV-1 prevention. Plos One. 2015 1;10(6):e0128857 DOI:10.1371/journal.pone.0128857 26061040 PMC4489588

[60] Decker MR, Peitzmeier S, Olumide A, Acharya R, Ojengbede O, Covarrubias L, et al Prevalence and health impact of intimate partner violence and non-partner sexual violence among female adolescents aged 15-19 years in vulnerable urban environments: a multi-country study. J Adolesc Health. 2014 12;55(6 Suppl):S58–67. DOI:10.1016/j.jadohealth.2014.08.022 25454004

[61] Thurston IB, Dietrich J, Bogart LM, Otwombe KN, Sikkema KJ, Nkala B, et al Correlates of sexual risk among sexual minority and heterosexual South African youths. Am J Public Health. 2014 7 11;104(7):1265–1269. 24832149 10.2105/AJPH.2013.301865 PMC4056197

[62] Gevers A, Mathews C, Cupp P, Russell M, Jewkes R Illegal yet developmentally normative: a descriptive analysis of young, urban adolescents’ dating and sexual behaviour in Cape Town, South Africa. BMC Int Health Hum Rights. 2013 1;13:31 DOI:10.1186/1472-698X-13-31 23841894 PMC3718713

[63] Peters RPH, Dubbink JH, van der Eem L, Verweij SP, Bos MLA, Ouburg S, et al Cross-sectional study of genital, rectal, and pharyngeal Chlamydia and gonorrhea in women in rural South Africa. Sex Transm Dis. 2014 9;41(9):564–569. DOI:10.1097/OLQ.0000000000000100 25118973

[64] Mavedzenge SN, Weiss HA, Montgomery ET, Blanchard K, de Bruyn G, Ramjee G, et al Determinants of differential HIV incidence among women in three southern African locations. J Acquir Immune Defic Syndr. 2011 9 1;58(1):89–99. 21654502 10.1097/QAI.0b013e3182254038

[65] Karim QA, Kharsany ABM, Frohlich JA, Werner L, Mashego M, Mlotshwa M, et al Stabilizing HIV prevalence masks high HIV incidence rates amongst rural and urban women in KwaZulu-Natal, South Africa. Int J Epidemiol. 2011 8 3;40(4):922–930. 21047913 10.1093/ije/dyq176 PMC3156366

[66] Noguchi LM, Richardson BA, Baeten JM, Hillier SL, Balkus JE, Chirenje ZM, et al Risk of HIV-1 acquisition among women who use diff erent types of injectable progestin contraception in South Africa: a prospective cohort study. Lancet HIV. 2015 7;2(7):e279–87. DOI:10.1016/S2352-3018(15)00108-3 26155597 PMC4491329

[67] Abdool Karim S, Ramjee G Anal sex and HIV tranmission in women. Am J Public Health. 1998;88(8):1265–1266. DOI:10.2105/AJPH.88.8.1265-a PMC1508299 9702169

[68] Ramjee G, Williams B, Gouws E, Van Dyck E, De Deken B, Karim SA The impact of incident and prevalent herpes simplex virus-2 infection on the incidence of HIV-1 infection among commercial sex workers in South Africa. J Acquir Immune Defic Syndr. 2005 7 1;39(3):333–339. 15980695 10.1097/01.qai.0000144445.44518.ea

[69] Ramjee G, Gouws E Prevalence of HIV among truck drivers visiting sex workers in KwaZulu-Natal, South Africa. Sex Transm Dis. 2002 1;29(1):44–49. DOI:10.1097/00007435-200201000-00008 11773878

[70] van Loggerenberg F, Dieter AA, Sobieszczyk ME, Werner L, Grobler A, Mlisana K HIV prevention in high-risk women in South Africa: condom use and the need for change. Plos One. 2012 1;7(2):e30669 DOI:10.1371/journal.pone.0030669 22363467 PMC3281865

[71] Dunkle KL, Beksinska ME, Rees VH, Ballard RC, Htun Y, Wilson ML Risk factors for HIV infection among sex workers in Johannesburg, South Africa. Int J STD AIDS. 2005 3;16(3):256–261. DOI:10.1258/0956462053420220 15829029

[72] Van Damme L, Ramjee G, Alary M, Vuylsteke B, Chandeying V, Rees H, et al Effectiveness of COL-1492, a nonoxynol-9 vaginal gel, on HIV-1 transmission in female sex workers : a randomised controlled trial. Lancet. 2002;360:971–977. 12383665 10.1016/s0140-6736(02)11079-8

[73] Simbayi LC, Kalichman SC, Jooste S, Cherry C, Mfecane S, Cain D Risk factors for HIV-AIDS among youth in Cape Town, South Africa. AIDS Behav. 2005 3;9(1):53–61. DOI:10.1007/s10461-005-1681-4 15812613

[74] Kalichman SC, Pitpitan E, Eaton L, Cain D, Carey KB, Carey MP, et al Bringing it home: community survey of HIV risks to primary sex partners of men and women in alcohol-serving establishments in Cape Town, South Africa. Sex Transm Infect. 2013 5;89(3):231–236. DOI:10.1136/sextrans-2012-050569 23241968 PMC3625822

[75] Pitpitan EV, Kalichman SC, Eaton LA, Sikkema KJ, Watt MH, Skinner D Gender-based violence and HIV sexual risk behavior: alcohol use and mental health problems as mediators among women in drinking venues, Cape Town. Soc Sci Med. 2012 10;75(8):1417–1425. DOI:10.1016/j.socscimed.2012.06.020 22832324 PMC3425436

[76] Kalichman SC, Simbayi LC, Cain D HIV transmission risk behaviours among HIV seropositive sexually transmitted infection clinic patients in Cape Town, South Africa. Eur J Public Health. 2010 4;20(2):202–206. DOI:10.1093/eurpub/ckp127 19726591 PMC2860715

[77] Kalichman SC, Simbayi L, Jooste S, Vermaak R, Cain D Sensation seeking and alcohol use predict HIV transmission risks: prospective study of sexually transmitted infection clinic patients, Cape Town, South Africa. Addict Behav. 2008 12;33(12):1630–1633. DOI:10.1016/j.addbeh.2008.07.020 18790575 PMC4271623

[78] Kiene SM, Simbayi LC, Abrams A, Cloete A, Tennen H, Fisher JD High rates of unprotected sex occurring among HIV-positive individuals in a daily diary study in South Africa: the role of alcohol use. J Acquir Immune Defic Syndr. 2008 10 1;49(2):219–226. 18769345 10.1097/QAI.0b013e318184559f PMC2631279

[79] Kalichman SC, Cain D, Eaton L, Jooste S, Simbayi LC Randomized clinical trial of brief risk reduction counseling for sexually transmitted infection clinic patients in Cape Town, South Africa. Am J Public Health. 2011 9;101(9):e9–17. DOI:10.2105/AJPH.2011.300236 PMC3154219 21778486

[80] Kiene SM, Christie S, Cornman DH, Fisher WA, Shuper PA, Pillay S, et al Sexual risk behaviour among HIV-positive individuals in clinical care in urban KwaZulu-Natal, South Africa. AIDS. 2006 8 22;20(13):1781–1784. 16931945 10.1097/01.aids.0000242827.05120.55

[81] Dunkle KL, Jewkes R, Nduna M, Jama N, Levin J, Sikweyiya Y, et al Transactional sex with casual and main partners among young South African men in the rural Eastern Cape: prevalence, predictors, and associations with gender-based violence. Soc Sci Med. 2007 9;65(6):1235–1248. DOI:10.1016/j.socscimed.2007.03.029 17560702 PMC2709788

[82] van Loggerenberg F, Mlisana K, Williamson C, Auld SC, Morris L, Gray CM, Establishing a cohort at high risk of HIV infection in South Africa: challenges and experiences of the CAPRISA 002 acute infection study. Plos One. 2008 1;3:e1954 DOI:10.1371/journal.pone.0001954 18414658 PMC2278382

[83] McFarlane M, St Lawrence JS Adolescents’ recall of sexual behavior: consistency of self-report and effect of variations in recall duration. J Adolesc Health. 1999 9;25(3):199–206. DOI:10.1016/S1054-139X(98)00156-6 10475496

[84] Graham CA, Catania JA, Brand R, Duong T, Canchola JA Recalling sexual behavior: a methodological analysis of memory recall bias via interview using the diary as the gold standard. J Sex Res. 2003 11;40(4):325–332. DOI:10.1080/00224490209552198 14735406

[85] Napper LE, Fisher DG, Reynolds GL, Johnson ME HIV risk behavior self-report reliability at different recall periods. AIDS Behav. 2010 2;14(1):152–161. DOI:10.1007/s10461-009-9575-5 19475504 PMC2814040

[86] Baggaley RF, Dimitrov D, Owen BN, Pickles M, Butler AR, Masse B, et al Heterosexual anal intercourse: a neglected risk factor for HIV?. Am J Reprod Immunol. 2013;69(SUPPL.1):95–105. DOI:10.1111/aji.12042 23279040 PMC3938911

[87] Philips A, Gomez G, Boily M-C, Garnett G A systematic review and meta-analysis of quantitative interviewing tools to investigate self-reported HIV and STI associated behaviours in low- and middle- income countries. Int J Epidemiol. 2010;39(6):1541–1555. DOI:10.1093/ije/dyq114 20630991

[88] Béhanzin L, Diabaté S, Minani I, Lowndes CM, Boily M-C, A-C L, et al Assessment of HIV-related risky behaviour: a comparative study of face-to-face interviews and polling booth surveys in the general population of Cotonou, Benin. Sex Transm Infect. 2013 11;89(7):595–601. DOI:10.1136/sextrans-2012-050884 23723251 PMC3800174

[89] Nnko S, Boerma JT, Urassa M, Mwaluko G, Zaba B Secretive females or swaggering males?. Soc Sci Med. 2004 7;59(2):299–310. DOI:10.1016/j.socscimed.2003.10.031 15110421

[90] Kalichman SC, Pinkerton SD, Carey MP, Cain D, Carey KB, Mwaba K Heterosexual anal intercourse and HIV infection risks in the context of alcohol. BMC Public Heal. 2011;11:807–819. DOI:10.1186/1471-2458-11-807 PMC3207968 21999574

[91] Duby Z, Colvin C Conceptualizations of heterosexual anal sex and HIV risk in five East African communities. J Sex Res. 2014 1;51(8):863–873. DOI:10.1080/00224499.2013.871624 24611445

[92] Bradley J, Rajaram S, Moses S, Gowda GC, Pushpalatha R, Ramesh BM, et al Female sex worker client behaviors lead to condom breakage: a prospective telephone-based survey in Bangalore, South India. AIDS Behav. 2012 5 11;17(2):559–567. 10.1007/s10461-012-0192-3 22576127

[93] Alexander M, Mainkar M, Deshpande S, Chidrawar S, Sane S, Mehendale S Heterosexual anal sex among female sex workers in high HIV prevalence states of India: need for comprehensive intervention. Plos One. 2014 1;9(2):e88858 DOI:10.1371/journal.pone.0088858 24586416 PMC3930672

[94] Geibel S Condoms and condiments: compatibility and safety of personal lubricants and their use in Africa. J Int AIDS Soc. 2013 7 9;16(1):18531. 23841994 10.7448/IAS.16.1.18531 PMC3708353

